# Evaluation of a COVID-19 IgM and IgG rapid test; an efficient tool for assessment of past exposure to SARS-CoV-2

**DOI:** 10.1080/20008686.2020.1754538

**Published:** 2020-04-14

**Authors:** Tove Hoffman, Karolina Nissen, Janina Krambrich, Bengt Rönnberg, Dario Akaberi, Mouna Esmaeilzadeh, Erik Salaneck, Johanna Lindahl, Åke Lundkvist

**Affiliations:** a Department of Medical Biochemistry and Microbiology, Zoonosis Science Center (ZSC), Uppsala University, Uppsala, Sweden; b Department of Medical Sciences, Infectious Diseases Uppsala University, Uppsala, Sweden; c Laboratory of Clinical Microbiology, Uppsala University Hospital, Uppsala, Sweden; d Department of Clinical Sciences, Swedish University of Agricultural Sciences, Uppsala, Sweden; e Department of Biosciences, International Livestock Research Institute, Hanoi, Vietnam

**Keywords:** COVID-19, sars-CoV-2, rapid test, IgM, IgG, diagnostics

## Abstract

COVID-19 is the most rapidly growing pandemic in modern time, and the need for serological testing is most urgent. Although the diagnostics of acute patients by RT-PCR is both efficient and specific, we are also crucially in need of serological tools for investigating antibody responses and assessing individual and potential herd immunity. We evaluated a commercially available test developed for rapid (within 15 minutes) detection of SARS-CoV-2-specific IgM and IgG by 29 PCR-confirmed COVID-19 cases and 124 negative controls. The results revealed a sensitivity of 69% and 93.1% for IgM and IgG, respectively, based solely on PCR-positivity due to the absence of a serological gold standard. The assay specificities were shown to be 100% for IgM and 99.2% for IgG. This indicates that the test is suitable for assessing previous virus exposure, although negative results may be unreliable during the first weeks after infection. More detailed studies on antibody responses during and post infection are urgently needed.

## Background

In late 2019, a cluster of cases of viral pneumonia of unknown aetiology was reported in Wuhan, Hubei Province, China. This new viral pneumonia, COVID-19 (Coronavirus Disease 2019), caused by the novel SARS-CoV-2 (Severe Acute Respiratory Syndrome Coronavirus-2), spread rapidly and developed into a global pandemic within three months from its initial detection [1–3]. Among other symptoms, those of COVID-19 often include fever and dry cough, which resemble respiratory illnesses caused by other viruses or bacteria [4–7]. Due to the overlapping manifestations, clinical diagnosis becomes problematic, especially during seasonal flu [8], why confirmation of COVID-19 depends on the detection of SARS-CoV-2 nucleic acid by reverse-transcriptase polymerase chain reaction (RT-PCR).

More than 1.26 million cases of COVID-19 in > 200 countries and territories, with more than 66.000 human deaths, have been reported ([9], 5 April 2020). Due to the limited testing in many geographical regions, it is clear that the total number of actual COVID-19 cases is much higher than the number of confirmed ones. In most of the confirmed COVID-19 cases, the patients are symptomatic showing fever, dry cough, and pneumonia, but also more atypical symptoms such as gastrointestinal manifestations as well as anosmia and ageusia. However, the virus has been detected in completely asymptomatic individuals, e.g. in a recent study from Italy, showing that 44% of the laboratory-confirmed cases lacked symptoms [10]. The knowledge concerning the actual number of asymptomatic vs. symptomatic infections is still limited. The same is true for the potentially growing herd immunity, where almost no data is available to date.

In the present study, we evaluated a commercially available assay, the COVID-19 IgG/IgM Rapid Test Cassette (Zhejiang Orient Gene Biotech Co Ltd, Huzhou, Zhejiang, China), developed for detection of SARS-CoV-2-specific antibodies.

## Material and methods

### Serum samples

Capillary blood samples or serum from 29 PCR-confirmed COVID-19 patients or convalescents, and capillary blood samples from 24 healthy volunteers, without any known history of SARS-CoV-2 infection/COVID-19, were included in the study. Anonymous blood donor sera from healthy adults (n = 80) and 20 serum samples from babies (6–12 months) collected before or during 2018 from the Uppsala Academic Hospital were used as negative controls. Clinical samples that had been deposited in Uppsala Biobank were anonymized and used in accordance with local ethical guidelines. They were all used with informed consent according to the Swedish Biobank law, which allows anonymized diagnostic patient samples to be used for purposes similar to those of the original sampling. The 29 samples from COVID-19 confirmed individuals, as well as the 100 negative controls and the 24 healthy volunteers were all from unique individuals. All samples were analyzed anonymously.

### COVID-19 IgG/IgM rapid test

The test was run according to the manufacturers instructions (COVID-19 IgG/IgM Rapid Test Cassette (whole blood/serum/plasma), Product/Model: GCCOV-402a, Lot: 2003242, Zhejiang Orient Gene Biotech Co Ltd, Huzhou, Zhejiang, China) [11]. Briefly, 5 µl of serum or one drop of capillary blood were added to the test slide, followed by 80 µl of the buffer provided in the kit. The results were read after 10 min (max 15 min), by the naked eye. Only tests in which the control line changed its color were regarded as valid (3 out of 156 (1.9%) cassettes did not function). If a line was observed for IgM and/or IgG, the test was considered positive. The intensity of the color was not judged.

## Results

### IgM and IgG reactivities in negative control samples

None of the 80 negative sera from healthy blood donors tested IgM positive in the assay, while one tested IgG positive (1/80, 1.25%, 95% confidence level: 0.03–6.77%) (Tables 1 and 2). The single IgG-positive sample was re-analyzed and remained IgG positive in the second test. None of the 20 serum samples from the 6–12 months old babies tested positive for either IgM or IgG.

**Table 1.** Comparisons of IgM results for 29 PCR-positive COVID-19 cases and 124 healthy individuals

	Cases	Healthy	Total
IgM positive	20	0	20
IgM negative	9	124	133
Total	29	124	153

**Table 2.** Comparisons of IgG results for 29 PCR-positive COVID-19 cases and 124 healthy individuals

	Cases	Healthy	Total
IgG positive	27	1	28
IgG negative	2	123	125
Total	29	124	153

### IgM and IgGreactivities in healthy volunteers

None of the 24 healthy volunteers, without any known history of SARS-CoV-2 infection/COVID-19, tested positive for IgM or IgG.

### IgM and IgGreactivities in PCR-confirmed COVID-19 patients

**Figure 1.**
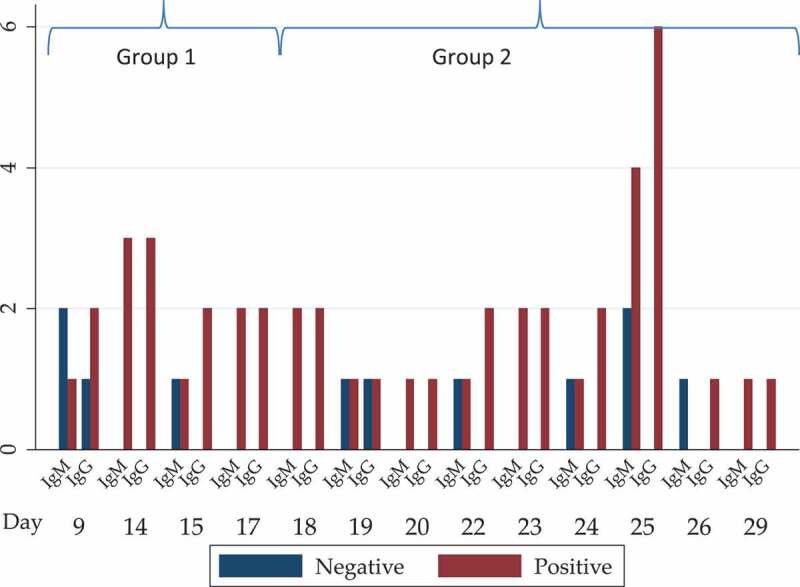
Number of PCR-positive cases positive or negative for IgM or IgG based on number of days after onset of COVID-19 symptoms

Altogether 20 of 29 (69%) samples from PCR-confirmed COVID-19 patients tested IgM positive and 27 tested (93.1%) IgG positive (Tables 1 and 2). When the patients were grouped into two groups depending on the time between onset of disease and testing, seven out of ten patients in the first group (9–17 days) and 13/19 patients in the second group (18–29 days) tested IgM positive. Nine out of ten patients in the first group (9–17 days) and 18/19 patients in the second group (18–29 days) tested IgG positive (Figure 1). There was no statistical difference between the two groups for neither IgM or IgG seropositivity. All samples that were IgM positive were also IgG positive.

### Assay sensitivity, specificity and accuracy

Based on the results described above and summarized in Tables 1 and 2, the assay showed a sensitivity of 69% (20/29) and 93.1% (27/29) for IgM and IgG, respectively. The assay showed an overall specificity of 100% (124/124) and 99.2% (123/124, 1 false positive) for IgM and IgG, respectively.

Using PCR-positive cases as true positives, the accuracy of the test was 94.1% (144/153) and 98.0% (150/153) for IgM and IgG, respectively. The positive and negative predictive values (the likelihood of being a case given a positive test result, and the likelihood of being healthy given a negative test result) for IgM were 100% (20/20) and 93.2% (124/133), respectively. For IgG, the corresponding values were 96.4% (27/28) and 98.4% (123/125).

## Discussion

In this study we evaluated a commercial rapid test for detection of SARS-CoV-2-specific IgM and IgG. For the evaluation, samples from COVID-19 cases, obtained during disease or convalescence and previously confirmed by PCR, were used as ‘true positives’. This means that in the PCR-positive cases for which antibodies may not yet had time to develop, or in potential cases with immune defects, it is possible that the negative IgM or IgG results were in fact true negatives. If this was the case for one or more of the included patients, the actual sensitivities should be higher, i.e. when evaluated only on samples known to contain detectable levels of SARS-CoV-2-specific IgM and/or IgG. For a more optimal evaluation of the assay sensitivity, a gold standard for SARS-CoV-2-specific antibodies would have been needed. This is, however, unfortunately not available to date.

According to the manufacturer, the specificity has been evaluated on 14 PCR-negative samples and was found to be 100% for both IgM and IgG, while the sensitivity evaluated on COVID-19 cases was calculated at 87.9% for IgM and 97.2% for IgG. The results by Li et al. [11] indicated an overall testing sensitivity of 88.7% and 90.6% specificity. Our results showed a lower sensitivity for IgM, a similar sensitivity for IgG, and specificities in between the results of the two evaluations.

A recent study on three Chinese COVID-19 cases found that seroconversions occurred between 7 and 12 days after the onset of symptoms [12]. However, larger studies on the detailed kinetics of the antibody responses (e.g. IgA, IgM, IgG, neutralizing antibodies) are now urgently needed for a better understanding of the dynamics of the immune response during COVID-19. The results of our study showed detectable IgM and IgG in some patients at day 9, while in other patients the seroconversion seems to occur later. The impact of early or late seroconversion on the case severity is not known, and must now be explored. Interestingly, there were no IgM positives that were not IgG positive. Generally, IgM is produced first, and later there is a switch towards IgG production [13], but studies on SARS-CoV suggest that IgM and IgG often develop around the same time [14,15]. Our results are in line with this (Figure 1), but more detailed studies on long-term sequential samples from patients are now needed. It may be worth looking into whether this is a problem with the test, or a constant finding within the immune response to SARS-CoV-2.

There were no false IgM positive samples, indicating a very high specificity of the test. One false positive IgG result was observed for one healthy adult blood donor. This sample was re-tested and the result was consistent, indicating a cross-reaction to another coronavirus. Serological cross-reactions have earlier been observed between SARS-CoV and SARS-CoV-2 [16]. There are other human coronaviruses (NL63, OC43, 229E, and HKU1) that are globally endemic or epidemic [17], and it may be possible that this reaction represented a cross-reaction due to a previous infection with one of those. Human CoV NL63 has been shown to use the same receptor, angiotensin-converting enzyme 2 (ACE2), as SARS-CoV and SARS-CoV-2 [18], which may indicate potential cross-reactive epitopes. How common the CoVs are as causative agents for ‘common colds’ is not known in detail, but there has been estimates that up to 20% of cases could be caused by CoVs [19].

The specificity and sensitivity for IgG detection of the rapid test evaluated here is well in line with those of a recently reported enzyme-linked immunosorbent assay (ELISA), which had a specificity and a sensitivity of 97.5% [20].

While this study showed a satisfactory performance of the rapid test, it is limited by being compared only to clinical cases and PCR-positivity, and as a next step, it is necessary to compare this assay to other serological tests. In contrast to Li et al. [11], we found less indications for using this test for clinical diagnosis. Nevertheless, it might contribute to detecting potential asymptomatic infections as well as getting a notion of the magnitude of the spread in different geographical areas, which might be a key to taking the appropriate decisions and policies forward. The high negative predictive value indicates that the rapid test will be useful for detecting past infections and possible immunity, which may be crucial for restoring social functions after lockdown.
