# We, the Publisher of *Infection Ecology & Epidemiology*, are issuing an Expression of Concern for the following article:

**DOI:** 10.1080/20008686.2025.2601432

**Published:** 2025-12-19

*Hoffman, T., Nissen, K., Krambrich, J., Rönnberg, B., Akaberi, D., Esmaeilzadeh, M., … Lundkvist, Å. (2020). Evaluation of a COVID-19 IgM and IgG rapid test; an efficient tool for assessment of past exposure to SARS-CoV-2.*

DOI: https://doi.org/10.1080/20008686.2020.1754538

After publication, concerns were raised regarding the lack of formal ethics approval for the research described. The Publisher’s investigation has found that multiple parties had previously reviewed these concerns, and these include the authors’ institution, the Ethics Review Appeals Board (ÖNEP), the Board for Investigation of Deviations from Good Research Practice (NUAF), and the district court.

The Ethics Review Appeals Board (ÖNEP) confirmed that prospective ethics approval would have been required for some of the samples included in this research; this was not obtained.

The district court closed the case with no charges being pressed and no prosecution initiated. The authors’ institution has indicated that no additional corrective action to the article was required. The Taylor and Francis Editorial policies require prospective ethics approval for human research studies such as that which is reported in the article. As this case cannot be resolved due to conflicting outcomes of the investigations conducted by the different parties, the article cannot be updated at this time.

In line with the Committee on Publication Ethics (COPE) guidelines and the Taylor and Francis Editorial policies, which require transparency in cases where there are ethical concerns but the Publisher’s investigations are inconclusive, we are issuing this Expression of Concern to alert readers and advise interpreting the information presented in the article with due caution.

The authors and institution have been notified about this Expression of Concern. Should further information come to light, the journal will reassess any concern raised.

